# EXIT (*ex utero* Intrapartum Treatment) to Airway Procedure for Twin Fetuses With Oropharyngeal Teratomas: Lessons Learned

**DOI:** 10.3389/fsurg.2020.598121

**Published:** 2020-10-26

**Authors:** Alice King, Sundeep G. Keswani, Michael A. Belfort, Ahmed A. Nassr, Alireza A. Shamshirsaz, Jimmy Espinoza, Joshua R. Bedwell, Deepak K. Mehta, Cara B. Doughty, Susan Margaret Leong-Kee, Julia B. Lawrence, Raphael C. Sun, Timothy C. Lee

**Affiliations:** ^1^Texas Children's Hospital, Houston, TX, United States; ^2^Baylor College of Medicine, Houston, TX, United States

**Keywords:** exit, teratoma, airway, twins, simulation

## Abstract

*Ex utero* intrapartum treatment (EXIT) to airway has been described as a safe method to secure challenging fetal airways while on placental support. Herein, we present a unique case of a monochorionic-diamniotic twin pregnancy where both fetuses presented with oropharyngeal tumors requiring airway securement on placental bypass. A multidisciplinary tabletop simulation was convened to allow for personnel coordination between multiple services, OR equipment allocation, and preparation for a range of possible clinical scenarios. A tabletop simulation was chosen for planning since this is a simulation methodology commonly used for preparation in acute, high intensity multidisciplinary situations such as disaster preparation, and allows for exploration of multiple potential scenarios when outcomes are uncertain. The twins were urgently delivered for decreased fetal movement and decelerations in Twin B at 28 weeks 6 days. Twin A was delivered *via* EXIT to airway while Twin B had debulking of the tumor on placental support, with subsequent airway securement through a tracheostomy. In conclusion, for complex fetal procedures, detailed pre-operative planning with tabletop simulation may be a useful tool in achieving successful patient outcomes.

## Background

*Ex utero* intra-partum procedures (EXIT) procedures in twin pregnancies represent an increased level of complexity and appropriate pre-operative planning and coordination is needed to help mitigate risk. To date, only sporadic case reports of EXIT procedures in twin pregnancies have been reported. All published case reports have described one affected and one normal fetus (1, 2). Some of the lessons learned from these case reports have focused on the ethical decision making in protecting the healthy fetus as well as the operative planning for EXIT to airway.

During the planning for complex case scenarios, one tool which can be used is tabletop simulation. The role of simulation is to facilitate multidisciplinary group debriefings through each possible emergency scenario to promote readiness for the actual event (3). Furthermore, one of the primary roles of these simulations is to allow all stakeholders to understand their role and responsibilities, and the interplay with other team roles. The most frequent venue for the use of this tool is in health disaster preparedness, as well as preparation for new processes, new healthcare spaces, and unique complex patients such as conjoined twins (4–6).

For this case report, we present how we used tabletop simulation in preparing for the unique case of a monochorionic diamniotic twin pregnancy requiring EXIT procedure for both fetuses due to an epignathic teratoma affecting one fetus and an oropharyngeal teratoma affecting the second fetus. The purpose of this report is 2-fold: one is to a discuss how tabletop simulation was used to facilitate planning and coordination of the case and the second goal is to describe the intra-operative lessons learned from this unique case where both fetuses required fetal intervention to obtain a successful outcome.

## Case Presentation

A 24 year old G2P0 woman presented to our fetal center at 22 weeks and 4 days with a monochorionic diamniotic twin pregnancy where twin A was found to have a small oral mass (2 × 1.3 cm) with cystic and solid components in twin A and a large solid, vascular and pedunculated mass (9 × 7 cm) in twin B. Fetal ultrasound showed normal Doppler studies in both twins with no signs of hydrops and no evidence of twin-twin transfusion syndrome initially. Fetal MRI demonstrated no evidence of airway obstruction in Twin A, however MRI confirmed a significant intraoral component in Twin B with substantial lateral deviation of the airway in the oropharynx, normal trachoesophageal complex, and fluid present in the stomach. At 24 weeks and 4 days, the fetuses developed Stage I TTTS. Fetoscopic-guided laser photocoagulation of placental anastomoses and amnioreduction (1L) was performed with survival of both fetuses. The patient was discharged home post-operative day 3.

Due to the anticipated complexity of patient delivery and that this was a unique case to our institution, we conducted a multidisciplinary, table-top simulation at 27 weeks gestation. This included orientation to simulation processes such as confidentiality, mutual respect, suspension of disbelief, and debriefing principles. The simulation facilitators were trained in debriefing with good judgment and PEARLS facilitation (7, 8). In brief, the scenarios which were discussed included (1) scheduled EXIT procedure at 32 weeks, (2) urgent EXIT procedure before 32 weeks and (3) emergent caesarian section. For each scenario and each phase of the EXIT procedure, detailed allocation of personnel and their responsibilities were clearly defined. Contingency plans for fetal airway management were delineated and necessary facilities and equipment were identified.

**Figure 1:**
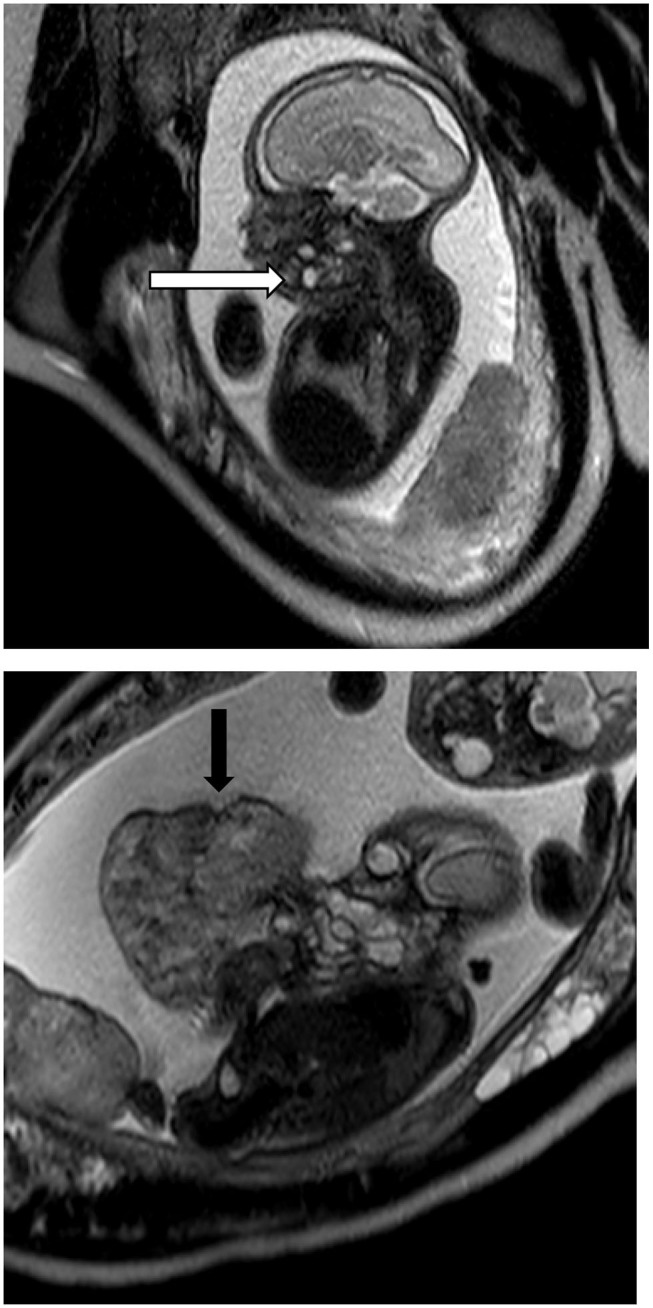
Fetal MRI of Twin A and Twin B. Twin A demonstrates a solid and cystic lesion at the floor of the mouth with obliteration of the oropharyngeal cavity (white arrow) while Twin B has the large epignathic teratoma arising from the oral cavity (black arrow).

A repeat fetal MRI was performed at 28 weeks, demonstrating substantial growth of oral lesions in both twins (see Figure 1). Twin A demonstrated obliteration of the fluid space between the tongue and palate with involvement of the entire floor of the mouth. The oral lesion of twin B had substantial increased with a predominantly solid components and significant vascularity. The pedunculated lesion occupied the oral cavity and floor of the mouth, extending along a pedicle to large 400 cc external component (Figure 1). Both fetuses demonstrated growth restriction with greater growth restriction in twin B.

At 28 weeks and 6 days, twin B was noted to have intermittent, severe decelerations with increase in painful maternal contractions refractory to magnesium sulfate. The decision to perform the EXIT procedure urgently was made. In the operating room, ultrasound demonstrated both twins in a cephalic position with Twin A more anterior and inferior. Twin A was partially delivered, exposing head and bilateral upper extremities. A direct laryngoscopy was performed while on uteroplacental circulation, demonstrating a large symmetric lesion involving the floor of the mouth. Fetal airway was established with a 2.5 uncuffed endotracheal tube *via* Seldinger technique over a rigid bronchoscope. Twin A was subsequently delivered and taken to its designated operating room where tube placement was confirmed, tube sutured at the gums and transition to neonatology team for further evaluation and resuscitation.

Twin B was subsequently partially delivered and noted to have a double nuchal cord. The pedunculated portion of the epignathic mass was bleeding and necrotic. This external portion was debulked using an endo-GIA stapler at the pedicle with good hemostasis. A direct laryngoscopy was attempted on placental circulation, but consistent with prenatal imaging, there was substantial involvement and distortion of the oral pharynx with inability to identify the airway. The nuchal cord was unable to be reduced with persistent fetal bradycardia therefore a blind nasal intubation was performed with clamping and division of the cord with transfer to the designated operating room for an emergency tracheostomy. A tracheotomy was performed and a 2.5 uncuffed endotracheal tube was passed into the tracheotomy. A red rubber was then passed in a retrograde fashion through the tracheotomy and out the mouth. An endotracheal tube was affixed to the red rubber and pulled antegrade *via* the oropharynx. The tracheotomy was repaired and the patient was subsequently transferred to the NICU.

## Discussion

Epignathic teratomas/oropharyngeal teratomas are rare lesions that can cause life-threatening neonatal airway obstruction. Advances in fetal imaging allow planning in order to improve outcome and limit hypoxia at delivery. The use of the EXIT procedure to facilitate airway securement in a controlled manner on placental bypass has been proposed as an optimal delivery strategy for patients with this type of pathology. However, the use of the EXIT procedure in twin gestations has increased complexity, requiring care and consideration of risks and benefits to mother and both fetuses. To date, the largest single center series reports three cases of EXIT procedure in twin pregnancies, all of which involve one affected twin and one normal twin (1). The conclusion by Garcia-Diaz et al. from their case series was that the risk should be borne by the affected fetus and priority to a safe outcome should be focused on the normal fetus.

This case was unique when compared to the known literature in that both fetuses required airway securement on placental support. When approaching their care, pre-operative planning was key in identifying the appropriate stakeholders in the operation. A tabletop simulation was planned which included twenty-nine people including pediatric surgery, otolaryngology (ENT), maternal fetal medicine (MFM), fetal anesthesia, fetal cardiology, pediatric operating room nursing staff, labor and delivery nursing staff and neonatology. A total of sixteen physicians and twelve nursing staff participated. As described in the case presentation, there were three case scenarios which were table top simulated. Scenario 2 and scenario 3 posed to be the most challenging in preparation. To prepare for scenario 2, the EXIT equipment was pre-positioned in the OR so that irrespective of the staffing or the time of day/night, the equipment would be ready for use. When discussing scenario number 3, scenario 3 would only occur if the less affected fetus (twin A) was to develop fetal distress and/or a maternal indication for immediate delivery such as placental abruption or precipitous delivery. Furthermore, if scenario 3 did happen, the in-house pediatric surgeon and on call pediatric anesthesiologist would attempt to stabilize the fetuses until the ENT service was in the delivery room. Prior to the simulation, the fetal surgery team had extensive discussion with the mother concerning her desires and the possible scenarios which were outlined in the simulation.

The planned EXIT procedure was scheduled for 32 weeks, recognizing limitations with twin gestations and prior fetoscopic laser therapy for TTTS. The goal was to have this performed in a scenario 1 situation. Delivery order of the twins was determined by their position, with both twins cephalic and twin A presenting more anterior and inferiorly. We identified the need for three adjoining operating suites (one for EXIT procedure, and one for each newborn). Space limitations within the rooms and at the operating room table were considered for coordination of necessary personnel during each phase of the EXIT procedure. In the table-top simulation, one of the key areas of planning was the role and responsibilities of personnel in the operating room and the operative field. The charge nurse was tasked with “crowd control” and all personnel in the room were required to have a role in the operation. In regards to personnel flow during the operation, the team was divided into one team for twin A and a second team for twin B. An ENT surgeon and pediatric surgeon were assigned to each team for airway securement. For the actual operation, uterine access was performed by two pediatric surgeons and two MFMs while two fetal cardiologists provided intra-operative fetal cardiac monitoring. On initial fetal exposure of twin A, the ENT surgeon replaced a MFM and an anesthesiologist came up to the field for peripheral vascular access.

Airway management and subsequent contingency plans were also discussed in the simulation. Direct laryngoscopy would be attempted first, followed by rigid bronchoscopy. If these failed, a laryngeal mask airway or nasotracheal intubation would be attempted, followed by fetal tracheotomy. Possible need for debulking of the tumor for twin B was anticipated and planned for in the EXIT operating room and for the operating room where twin B would go to after delivery. These rooms were equipped with the appropriate surgical stapling devices as well as handheld energy devices (Ligasure, Impact). As it turned out, the airway for twin A was secured with rigid bronchoscopy on placental support. Twin B did have the pedunculated mass resected on placental support however developed fetal bradycardia due to a nuchal cord which was unable to be released.

Another intra-operative lesson learned in this case is that the fetal cardiologists have to be extremely facile with obtaining the necessary windows to monitor the cardiac status during a twin EXIT. One technical issue which was encountered was that each ultrasound was found to interfere with the other ultrasound due to crossing of the ultrasound waves. This technical limitation narrowed the window to monitor fetal cardiac function and was an additional challenge in an already tight working field. The role of the fetal cardiologist is essential in these cases since they identified the fetal bradycardia in twin B due to the nuchal cord and allowed us to deliver and secure the airway in the adjacent operating room.

Finally, in each respective OR suite, there were teams which were receiving real time updates by video streaming of the EXIT onto their monitors in the room. This allowed for preparation and also situational awareness for when the babies were delivered. This was especially critical for twin B since the patient was delivered without a secure airway and needed an immediate tracheostomy in the adjacent operating room.

In conclusion, the EXIT procedure is a safe mode of delivery for patients identified with obstructing oropharyngeal lesions with potential risk of neonatal airway obstruction. The need to perform an EXIT in a twin gestation whom both fetuses are affected adds complexity that requires planning and coordination of a large multidisciplinary group. Tabletop simulation may be used as a tool in the surgical planning for such a case and as a possible planning tool for future complex operations.

## Data Availability Statement

The original contributions presented in the study are included in the article/supplementary material, further inquiries can be directed to the corresponding author/s.

## Ethics Statement

Ethical review and approval was not required for the study on human participants in accordance with the local legislation and institutional requirements. The patients/participants provided their written informed consent to participate in this study. Written informed consent was obtained from the individual(s) for the publication of any potentially identifiable images or data included in this article.

## Author Contributions

AK was involved in the care of this patient and played a significant role in drafting and revising the manuscript. SK played a significant role in the care of this patient, planning of the procedure, and in the drafting and revision of the case report. MB was involved in the operative intervention and care for this patient and in the revision of this paper. AN was significantly involved in the planning of the procedure and in the drafting and revision of the case report. AS and JE were significantly involved in the care of the patient, planning of the procedure, and in the drafting and revision of the case report. JB and DM had significant roles in the planning of the procedure and in the drafting and revision of the case report. CD, JL, and SL-K were involved in the operative planning for the procedure and revision of this manuscript. RS played a significant role in the planning of the procedure and in the drafting and revision of the case report. TL was significantly involved in the care of this patient, planning of the operation, and drafting and revision of this manuscript. All authors contributed to the article and approved the submitted version.

## Conflict of Interest

The authors declare that the research was conducted in the absence of any commercial or financial relationships that could be construed as a potential conflict of interest.
